# Early modulation of the gut microbiome by female sex hormones alters amyloid pathology and microglial function

**DOI:** 10.1038/s41598-024-52246-6

**Published:** 2024-01-21

**Authors:** Piyali Saha, Ian Q. Weigle, Nicholas Slimmon, Pedro Blauth Poli, Priyam Patel, Xiaoqiong Zhang, Yajun Cao, Julia Michalkiewicz, Ashley Gomm, Can Zhang, Rudolph E. Tanzi, Nicholas Dylla, Ayman Al-Hendy, Sangram S. Sisodia

**Affiliations:** 1 Department of Neurobiology, The University of Chicago, Chicago, IL USA; 2 School of Biomedical Sciences, UT Southwestern Medical Center, Dallas, TX USA; 3 Center for Genetic Medicine, Northwestern University, Chicago, IL USA; 4 Genomic Facility, The University of Chicago, Chicago, IL USA; 5 Department of Physiology and Biophysics, The University of Illinois, Chicago, IL USA; 6 Department of Neurology, Harvard Medical School, Boston, MA USA; 7 Duchossois Family Institute, The University of Chicago, Chicago, IL USA; 8 Department of Obstetrics and Gynecology, The University of Chicago, Chicago, IL USA

**Keywords:** Neuroscience, Molecular neuroscience

## Abstract

It is well-established that women are disproportionately affected by Alzheimer’s disease. The mechanisms underlying this sex-specific disparity are not fully understood, but several factors that are often associated-including interactions of sex hormones, genetic factors, and the gut microbiome-likely contribute to the disease's etiology. Here, we have examined the role of sex hormones and the gut microbiome in mediating Aβ amyloidosis and neuroinflammation in APPPS1-21 mice. We report that postnatal gut microbiome perturbation in female APPPS1-21 mice leads to an elevation in levels of circulating estradiol. Early stage ovariectomy (OVX) leads to a reduction of plasma estradiol that is correlated with a significant alteration of gut microbiome composition and reduction in Aβ pathology. On the other hand, supplementation of OVX-treated animals with estradiol restores Aβ burden and influences gut microbiome composition. The reduction of Aβ pathology with OVX is paralleled by diminished levels of plaque-associated microglia that acquire a neurodegenerative phenotype (MGnD-type) while estradiol supplementation of OVX-treated animals leads to a restoration of activated microglia around plaques. In summary, our investigation elucidates the complex interplay between sex-specific hormonal modulations, gut microbiome dynamics, metabolic perturbations, and microglial functionality in the pathogenesis of Alzheimer's disease.

## Introduction

Alzheimer's disease (AD) is a neurodegenerative disorder characterized by cognitive decline, memory loss, and behavioral alterations. AD disproportionately impacts women compared to men^1^, but the molecular and cellular mechanisms that underlie this significant sex disparity are poorly understood. Additionally, genetic and epigenetic factors may also contribute to the sex differences in AD. For example, the principal genetic risk factor for late-onset AD, apolipoprotein E4, confers greater risk in females^2,3^. Furthermore, sex-specific epigenetic modifications may influence gene expression patterns and contribute to the differential susceptibility to AD between males and females^4^.

The pathophysiological changes associated with AD are known to begin years before the onset of cognitive and behavioral symptoms^5,6^. In women, this suggests that pathogenic processes may occur in the brain during the reproductive stage, before the cessation of ovarian function. While ovarian functions have been shown to impact neural networks and exhibit cyclic fluctuations^7,8^, it remains unclear whether these functions affect the underlying pathophysiological substrates that contribute to cognitive impairments in dementia.

We previously demonstrated that antibiotic (ABX)-mediated dysbiosis of the gut microbiome in the APPswe/PS1∆E9 and APPPS1-21 mouse models of Aβ amyloidosis led to reductions in Aβ deposition through altered microglial phenotypes in male, but not female mice^9,10^. Microglia, the innate immune cells in the brain, express risk genes associated with late-onset AD^11^. Moreover, and by mechanisms presently unclear, the gut microbiome influences microglial development in a sex-specific manner^12^. Notably, sex hormones have been shown to modulate the composition of the gut microbiota and vice-versa^13^. Extending these findings, we asked whether alterations of circulating female sex hormones in early life can modulate Aβ amyloidosis, microglial phenotypes and the composition of the gut microbiome.

Our studies reveal that postnatal ABX leads to an elevation in circulating estradiol and that lowering of plasma estradiol by ovariectomy (OVX) at an early age resulted in a significant decrease in Aβ plaque pathology and pronounced alterations in the gut microbiome composition in young mice. Estradiol supplementation of ovariectomized mice lead to a restoration of plaque burden and an altered gut microbiome. Moreover, OVX treatment decreased the levels of disease-associated “MGnD”-type^14^ microglia that are plaque localized, while ovariectomized mice supplemented with estradiol exhibited a restoration of plaque-localized MGnD-type microglial cells. RNAseq studies confirmed that the fluctuations in estradiol concentrations were associated with changes in levels of mRNAs encoding proteins involved in metabolic processes and immune profiles in cortical tissue from female mice. Thus, our studies highlight the multifaceted role of estradiol in the regulation of Aβ burden, the gut microbiome, and innate immunity in a mouse model of Aβ amyloidosis.

## Results

### Elevated circulating levels of estradiol in antibiotic-treated female APPPS1-21 mice

**Figure 1:**
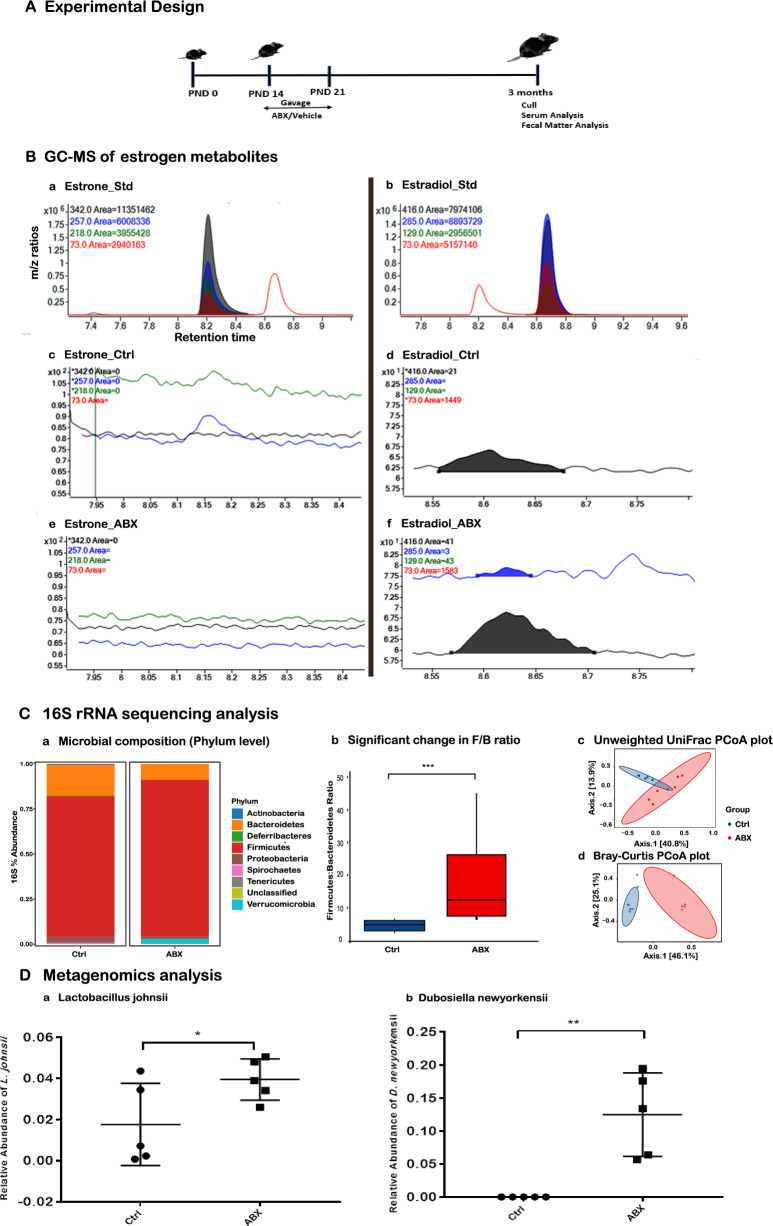
The effects of ABX treatment on estrogen metabolism and associated changes in the gut microbiome were investigated in APPPS1-21 female mice. (**A**) Timeline of experimental design depicting vehicle (control) and antibiotics (ABX) gavage in mice from postnatal day 14 to 21, followed by sacrifice for serum and fecal matter analysis. (**B**) Representative GC–MS chromatograms display plasma estrogen metabolites, with the following details: (**a**) Estrone_standard—used as a reference for establishing retention time and fragmentation pattern, with the ion 342 quantified by the raw area under the curve; (**b**) Estradiol_standard—also serving as a reference for retention time and fragmentation, with ion 416 used for quantification; (**c**) Estrone_control—showing no detectable estrone; (**d**) Estradiol_control—illustrating baseline estradiol levels; (**e**) Estrone_ABX—indicating no estrone detected; and (**f**) Estradiol_ABX—demonstrating elevated estradiol levels in ABX-treated mice compared to the vehicle-treated group. Calculations based on the area under the curve show an increase in estradiol levels in ABX-treated mice compared to the vehicle-treated group. This data was obtained from pooled serum samples of 5–8 mice per group, and therefore, no statistical analysis was conducted. (**C**) The 16S rRNA sequencing analysis (n = 6 mice/group) is presented as follows: (**a**) Bar plots reveal phylogenetic differences between ABX-treated and vehicle-treated mice, with a focus on the abundances of Firmicutes and Bacteroidetes. (**b**) In APPPS1 mice, a significant increase in the Firmicutes/Bacteroidetes (F/B) ratio is observed in ABX-treated mice compared to those treated with the vehicle (Wilcoxon test, *p* = 0.0079). (**c**) An Unweighted-UniFrac PCoA plot, accounting for 40.8% of the variance (permanova test, *p* = 0.02), demonstrates a significant clustering effect. (**d**) A Bray–Curtis PCoA plot, capturing 46.1% of the variance (Permanova test, *p* = 0.002), reveals a distinct and significant cluster. (**D**) The bar graph, generated through a high-throughput metagenomics analysis (n = 5 mice/group), illustrates the increased abundance of the bacterial species *Dubosiella newyorkensis* and *Lactobacillus johnsii* in the ABX group compared to the control group in Thy1-APPPS1 female mice (unpaired Student’s t-test: t[7]  = 1.95, *p* = 0.0461). The increase in these species within the ABX group is speculated to lead to elevated levels of estradiol. These bacteria are associated with estrogen metabolism in the Firmicutes phylum. Data are presented as mean ± SEM, with the following significance levels: *, *p* < 0.05; **, *p* < 0.01; ***, *p* < 0.001.

In preceding efforts we reported that APPPS1-21 mice treated with a high dose of ABX cocktail postnatally (P14-P21) led to gut microbiome dysbiosis^10^ that was associated with a reduction in Aβ deposition in 3-month-old male mice, an effect not observed in their female counterparts^10^. Despite marked changes in the gut microbial composition of female mice treated with ABX, compared with animals treated with a vehicle^10^, the mechanisms underlying the absence of changes in Aβ deposition in females remained unclear. According to Plottel and Blaser^13^,“it is well known that estrogens produced in the ovaries, adrenal glands, and adipose tissue circulate in the bloodstream and first undergo metabolism in the liver, where estrogens and their metabolites are conjugated. Conjugated estrogens are eliminated from the body by metabolic conversion to water-soluble molecules that are excreted in urine or in bile into the feces. The inactive conjugated estrogens excreted in the bile are deconjugated by bacterial species in the gut (the “estrabolome”) by microbially synthesized β-glucuronidase (gmGUS) leading to estrogen reabsorption into the circulation”^13^. Hence, we speculated that ABX-mediated dysbiosis might affect the estrabolome in a manner that elevates circulating estrogen. To test this hypothesis, we employed ultrasensitive GC–MS approaches to examine the levels of estrogen metabolites, estrone and estradiol, compounds considered the most biologically active among all estrogen metabolites. We now demonstrate that the levels of plasma estradiol were elevated in ABX-treated female mice (n = 5–7 mice/group pooled plasma sample) [Fig. 1A] at the time of sacrifice compared to the levels in plasma of vehicle-treated mice (control). In GC–MS analysis, compound fragmentation leads to the creation of distinct ionized fragments, each characterized by a specific mass-to-charge ratio (m/z), which forms a unique pattern akin to a molecular 'fingerprint'. In our study, the colored lines on the chromatograms correspond to these fragments for estrone [Fig. 1B, panel a] and estradiol [Fig. 1B, panel b]. Specifically, the estrone fragments are indicated as follows: 342 (black), 257 (blue), 218 (green), and 73 (red). Similarly, for estradiol, the fragmentation pattern includes 416 (black), 285 (blue), 129 (green), and 73 (red). This color-coding in the chromatograms facilitates precise identification and quantification of the compounds, thereby enhancing the accuracy and specificity of our analysis. Figure 1A, panel a, represents the estrone standard, employed for measuring the retention time and fragmentation pattern. The ion with an m/z ratio of 342 was used to measure the raw area under the curve. Notably, estrone was not detected in Fig. 1A panel c (control) and Fig. 1A panel e (ABX). Conversely, Fig. 1A panel b represents the estradiol standard, also used for establishing retention time and fragmentation pattern. The ion with an m/z of 416, detected in plasma samples from both the control (panel d) and ABX-treated (panel f) groups, was used for measurement. In our analysis, the ion with an m/z, detected in plasma samples from both the control and ABX-treated groups, was selected for quantitative measurement. In this context, the ‘Area Under the Curve’ (AUC) is a crucial metric used in GC–MS analysis. AUC refers to the area under the plot of the compound's intensity against time or another relevant variable in the chromatogram. It is directly proportional to the concentration of the compound in the sample.

To elaborate, in Fig. 1A, panel d, representing the control group, the AUC for estradiol is quantified as 21. This value indicates the total concentration or presence of estradiol over the observed time period in the control group, as determined by integrating the area under its peak in the chromatogram. Conversely, Fig. 1A, panel f, depicting the ABX-treated group, shows an estradiol AUC of 41. This higher value suggests a greater presence or concentration of estradiol in the ABX-treated group compared to the control. Thus, these AUC values (21 and 41) are indicative of the relative amounts of estradiol detected in each group, providing a quantitative comparison of estradiol levels between the control and ABX-treated samples using GC–MS analysis. Similarly, earlier studies have shown that a broad-spectrum antibiotic combination [sulfamethoxazole and trimethoprim] elevates the plasma concentration of ethinyl estradiol^15^. Importantly, ABX treatment in female APPPS1-21 mice resulted in substantial alterations in microbiome diversity [Fig. 1C, panel a, c, d] and a significant increase in the Firmicutes/Bacteroidetes (F/B) ratios compared to vehicle-treated mice at the time of sacrifice (n = 6 mice/group, p = 0.008) [Fig. 1C, panel b]. Changes in F/B ratios have been reported to affect the composition of the estrobolome^13^. With the aid of comprehensive metagenomic studies, we identified two bacterial species, *Dubosiella newyorkensis* (n = 6 mice/group, *p* = 0.002) and *Lactobacillus johnsii* (n = 6 mice/group, *p* = 0.04) within the Firmicutes family that were significantly elevated in the ABX group and are known to express gut microbial β-glucuronidase (gmGUS) [Fig. 1D]. We suggest that elevated levels of these species in the ABX group would lead to elevated levels of estradiol.

### Ovariectomy of female APPPS1-21 mice leads to significant decrease in amyloid plaque burden while estradiol supplementation restores pathology

**Figure 2:**
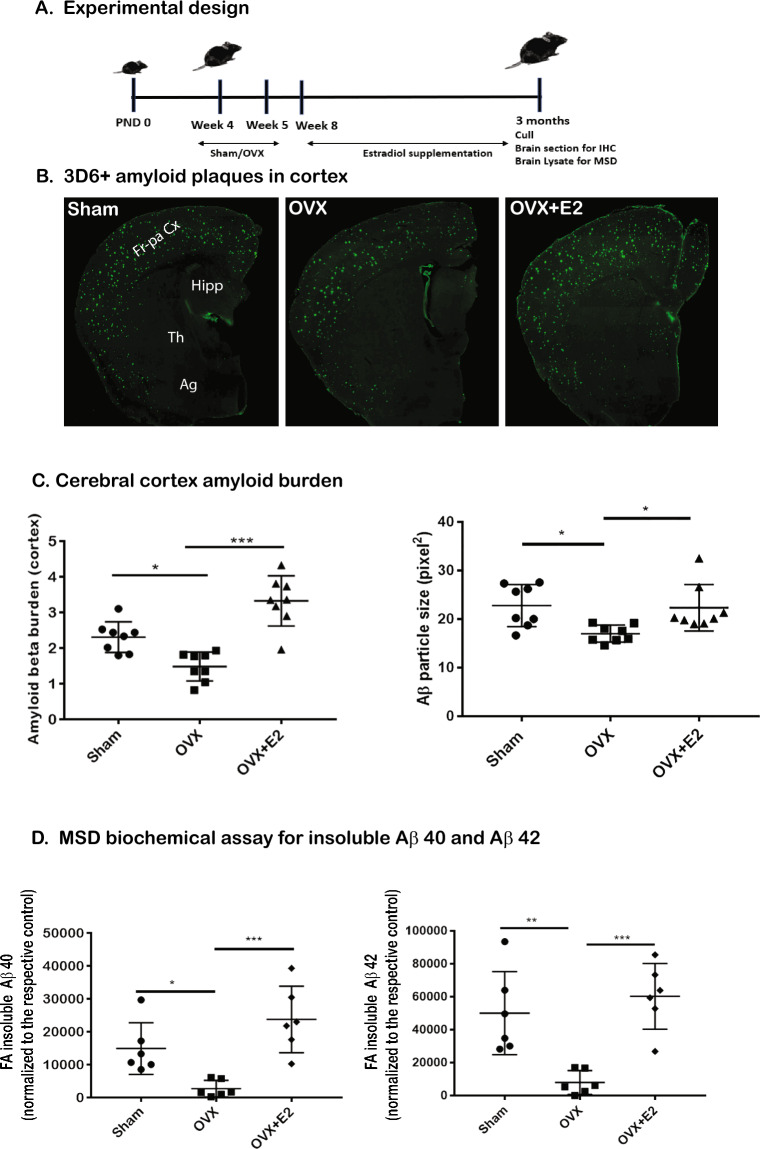
Impact of OVX and Estradiol Supplementation on Aβ Pathology in APPPS1-21 Female Mice. (**A**) Experimental design represented on a timeline showing Sham (control), ovariectomy (OVX), and OVX with estradiol (E2) supplementation in mice, which were used for brain sectioning for immunohistochemistry (IHC) and brain lysate analysis using Meso Scale Discovery (MSD) post-sacrifice. (**B**) The figure displays representative images of Aβ plaque burden and plaque sizes in the cortex of three groups: Sham-operated, OVX, and estradiol-supplemented mice (OVX + E2), using the anti-Aβ monoclonal antibody, 3D6 (scale = 20 microns); Fr-pa Cx = Fronto-parietal Cortex, Hipp = Hippocampus, Th = Thalamus, Ag = Amygdala (n = 8 mice/group). (**C**) The Aβ plaque burden and plaque size were quantified using threshold-limited particle analysis of 3D6-positive staining from six sections per case, and the results were expressed relative to the total cortical area of each slice. Notably, the OVX mice show reduced cortical Aβ levels and smaller plaque sizes compared to the Sham group. This reduction in Aβ levels and plaque size is reversed in mice that received estradiol supplementation (OVX + E2) (8 mice/group). (**D**) Biochemical assays assessing the levels of both soluble and insoluble forms of Aβ1-40 and Aβ1-42 in frozen ventral cerebral cortex tissues (n = 6 mice/ group) were conducted. These assays corroborate the immunohistochemical findings, demonstrating a significant decrease in the insoluble forms of Aβ1-40 and Aβ1-42 in the OVX mice. This reduction is offset in the mice that were supplemented with estradiol (statistical analysis with significance at *P* < 0.05). Data are presented as mean ± SEM, with the following significance levels: *, *p* < 0.05; **, *p* < 0.01; ***, *p* < 0.001.

To assess the potential role of estrogen in modulation of Aβ deposition, we performed OVX of female APPPS1-21 mice at the age of 4–5 weeks, then sacrificed the animals at 3 months, a time point when Aβ deposition is significant in the cortex [Fig. 2A]. We validated the successful execution of OVX by examining three key indicators: uterine weight, body weight, and estrogen/progesterone ratios (Fig. S2A). Ovarian hormones, such as estradiol and progesterone, play pivotal roles in maintaining energy balance, body composition, and glucose homeostasis^16^. OVX in APPPS1-21 female mice leads to a significant increase (n = 12 mice/group, p = 0.007) in body weight compared with the sham-operated group (Fig. 2Sa), indicating a potential impact on metabolic processes. OVX also results in a significant reduction (n = 7–8, *p* < 0.001) in uterine weight (Fig. 2Sb), indicating hormonal regulation loss^17^. As predicted^17^, OVX causes a significant drop in circulating estradiol /progesterone levels (n = 4–5 mice/group, *p* = 0.005) compared with the sham-operated group (Fig. 2Sc). Similarly, estradiol supplementation in the drinking water of OVX-treated mice reverses all the OVX-related decreases in estradiol/progesterone levels (n = 4–5 mice/group, *p* = 0.003) seen in OVX mice (Fig. 2SA). Thus, we confirm that APPPS1-21 mice subject to OVX leads to decreased circulating female sex hormone levels that can be reversed by estradiol supplementation.

To examine Aβ deposition in the brains of OVX and OVX-estradiol cohorts, we performed immunohistochemistry (IHC) with the Aβ-specific monoclonal antibody, 3D6^10,18,19^. We observed significant Aβ deposition in the cortex of Sham-operated mice, but OVX-treated female mice exhibited reduced cortical Aβ deposits and smaller plaque sizes compared to the Sham group (n = 8 mice/group, *p* = 0.01) (Fig. 2B and C). This reduction was offset in OVX-treated mice supplemented with estradiol (n = 8 mice/group, *p* < 0.001) (Fig. 2B and C). To validate these IHC studies, we performed biochemical assays to assess the levels of insoluble forms of Aβ1–40 and Aβ1–42 in frozen ventral cerebral cortex tissues using the mesoscale discovery (MSD) platform [Fig. 2D]. The levels of insoluble (formic acid (FA)-soluble) Aβ1–40 (n = 6 mice/group, *p* = 0.03) and Aβ1–42 (n = 6 mice/group, *p* = 0.004) were significantly reduced in extracts from brains of mice subject to OVX compared with the levels in Sham-operated controls. MSD assays confirmed that levels of FA-insoluble Aβ species returned to levels that were present in the brains of Sham-treated animals (n = 6 mice/group, *p* < 0.0007). Finally, it was important to establish that OVX or estradiol supplementation of OVX-treated animals had no impact on the steady-state levels of transgene-encode human *APP751* transgene in the brains of APPPS1-21 mice. For this analysis, we performed Western blot analysis of cortical extracts using a human APP-specific 6E10 antibody (Biolegend) that detects an epitope within amino acids “DAEFRHDSGYEVHHQ” in the human APP sequence that differs from the analogous sequence in mouse APP (“DAEFGHDSGFEVRHQ”) (Fig. 2SB). These studies failed to reveal any impact of OVX or OVX + estradiol supplementation on steady-state levels of human APP751 in the cortex (n = 3 mice/group, *p* = 0.2). Collectively, these findings demonstrate that in female APPPS1-21 mice, OVX resulted in a significant reduction in Aβ amyloidosis and that estradiol plays a role in modulating pathology in these animals.

### Circulating levels of estradiol correlate with alterations in the gut microbiome profile/composition in female mice

**Figure 3:**
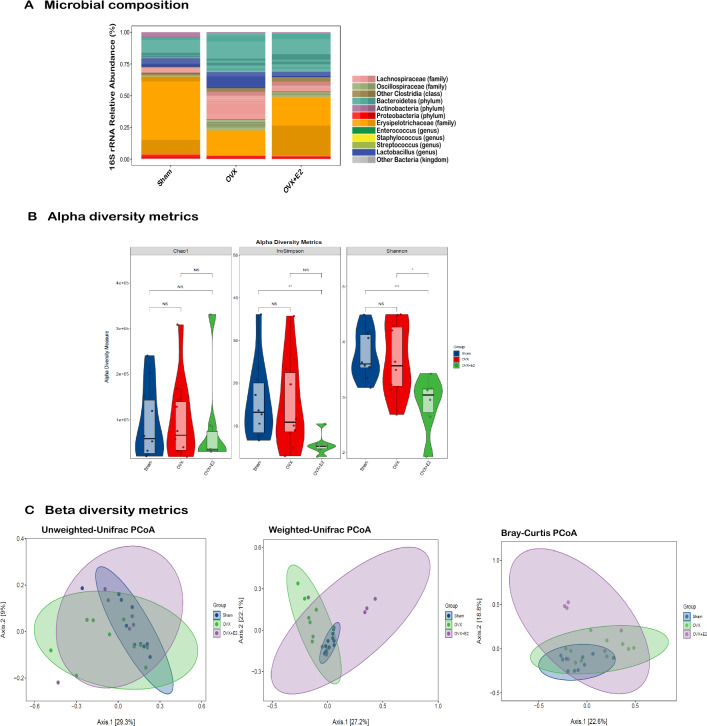
Impact of circulating levels of estradiol on the gut microbiome profile in female APPPS1-21 mice. (**A**) Bar chart delineating the differential abundance of distinct phylogenetic taxa across Sham, OVX, and OVX + E2 murine cohorts. (**B**) Alpha diversity analysis indicating an increased microbial diversity in OVX mice with low circulating estradiol levels compared to Sham mice with high levels of plasma estradiol (*p* < 0.001***). (**C**) Principal component analysis (PCA) plot representing the distinct clustering of OVX, Sham, and OVX + E2 samples based on plasma estradiol levels, illustrating a separate grouping of mice with low, intermediate, and high estradiol levels. The overall results suggest a significant regulatory influence of estradiol on the composition of the gut microbiota, underscoring the differences between OVX, Sham, and OVX + E2 mice. Data are represented as mean ± SEM; *, *p* < 0.05; **, *p* < 0.01, ***, *p* < 0.001. Sample size: n = 5–8 mice/group.

To investigate the impact of circulating levels of estradiol on the microbiome profile in female mice, we analyzed the composition of the gut microbiota in three groups of mice with varying estradiol levels: namely, the Sham group with normal physiological estradiol levels, the OVX group with lower estradiol levels, and the OVX + E2 mice supplemented with 5µg/ml of estradiol. We found that circulating levels of estradiol had a profound effect on the microbiome profile. The phylogenetic composition of gut microbiota exhibited distinct profiles between the Sham, OVX, and OVX + E2 female mouse cohorts (Fig. 3A). This was also observed in the principal coordinate analysis (PCoA) plot, where samples from mice with high plasma estradiol levels significantly clustered (n = 5–8 mice/group, *p* < 0.001) separately from those with low estradiol levels (Fig. 3C). Moreover, alpha diversity analysis that measure species richness and abundance revealed (Shannon and Inverse Simpson indices) that mice with low circulating estradiol levels had increased microbial diversity (n = 5–8 mice/group, *p* < 0.001) compared with mice with high levels of plasma estradiol (Fig. 3B). These results indicate that circulating levels of estradiol influences the microbiome profile in female mice, as expected^13^. The observed differences in phylogenetic composition between Sham, OVX and OVX + E2-treated (n = 5–8 mice/group, *p* < 0.001) mice suggests a regulatory role of estradiol in shaping the composition of the gut microbiota.

### Modulation of cortical transcripts levels by estradiol

**Figure 4:**
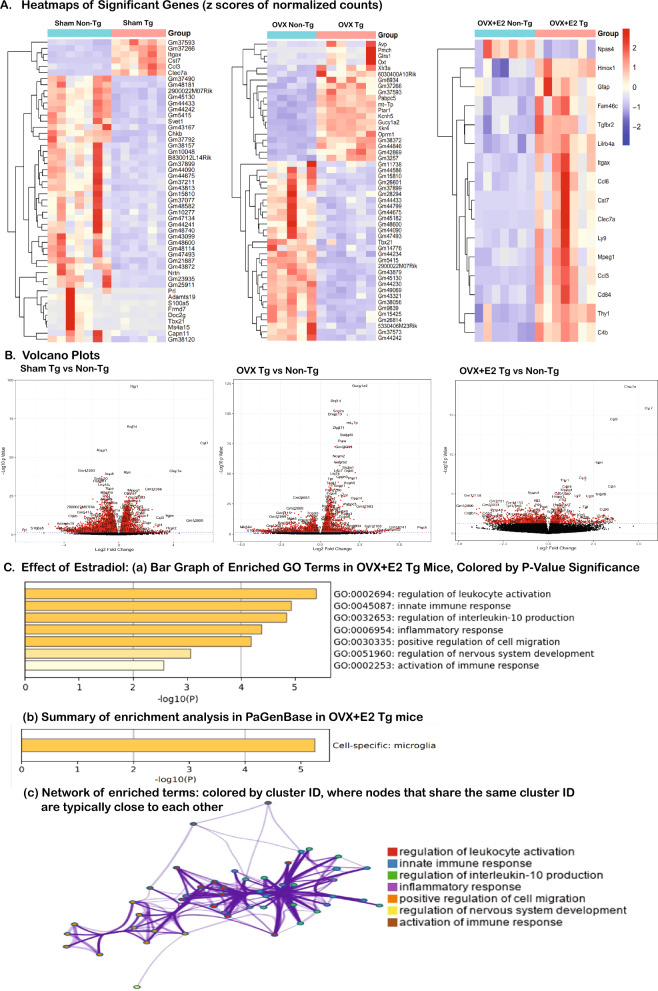
Cortical Transcriptomic RNA-seq Analysis in Non-Transgenic (non-Tg) vs Transgenic (Tg) Mice. (**A**) Heatmaps of significant gene expression variations in Sham (control), ovariectomized (OVX), and OVX supplemented with estradiol (OVX + E2) groups, highlighting differences between non-Tg and Tg mice using z-scores of normalized counts. (**B**) Volcano plots, emphasizing differential gene expressions across these groups. (**C**) Metascape software (current version v3.5.20240101), an advanced tool for comprehensive gene list annotation and analysis, leveraging resources like STRING, EggNog, and WikiPathways for enriched Gene Ontology (GO) term analysis (panel **a**) in OVX + E2 Tg mice categorized by P-value significance, illustrating the specific cell-type modulated by estradiol (panel **b**) and providing a network (panel **c**) of enriched terms, showing the interconnections between different GO terms and pathways. Data represent n = 6 mice/group, with statistical significance noted at *p* < 0.05.

In order to elucidate the effects of ovariectomy and estradiol supplementation on cortical gene expression, we conducted a comprehensive bulk RNA sequencing (RNA-seq) study on RNA isolated from the dorsal cerebral cortex of female APP/PS1-21 transgenic (Tg) mice and their non-transgenic (non-Tg) counterparts that were subject to Sham, OVX, or OVX + E2 treatments. The meta-analysis of the RNA-seq data revealed significant transcriptional differences between Sham, OVX, and OVX + E2 Tg versus non-Tg groups (n = 6 mice/group, employing a false discovery rate [FDR]-adjusted *p*-value < 0.05, as delineated in Fig. 4A). The heatmap in Fig. 4A graphically illustrates the absolute log fold changes (z-scores of normalized counts), accentuating the top upregulated and downregulated genes. Remarkably, in the Sham Tg mouse group, a distinctive upregulation of a cluster of microglia-specific genes that are associated with MGnD-type microglia (Itgax, Cst7, Ccl3, Clac7a) was observed compared with the non-transgenic mouse group. This pattern was further complemented by significant alterations in the expression of other genes, including those annotated as Gm37593, Gm37266, Gm48181, etc. The absence of increased expression in these genes post-OVX offers valuable insights into the hormone-dependent mechanisms governing microglial functions and potentially underscores the neuroprotective roles of ovarian hormones in the cerebral cortex. Subsequently, estradiol supplementation in OVX mice elicited a pronounced restoration of expression of Clec7a, Cst7, Ccl6, Itgax, and others such as Lilrb4 (associated with the innate immune system and antigen processing), Ly9 (a SLAM family receptor involved in immune regulation), Mpeg1 (a macrophage-expressed gene implicated in immune function), Cd84 (another SLAM family receptor), and C4b (a complement system component). To corroborate these findings, volcano plot analysis (Fig. 4B) was employed and highlights statistically significant alterations in terms of fold changes and P-values. Intriguingly, a similar set of microglia-specific genes (namely, Clec7a, Cst7, Ccl3, Ccl6, Itgax) was found to be upregulated upon estradiol supplementation of OVX-treated Tg mice compared with non-Tg mice. Further validation through gene ontology (GO) pathway analysis (Fig. 4C, panel a) revealed that immune regulatory pathways (Fig. 4C, panel c) were prominently influenced by estradiol treatment, with pathway enrichment studies indicating a significant impact on microglia-specific cell types (Fig. 4C, panel b).

**Figure 5:**
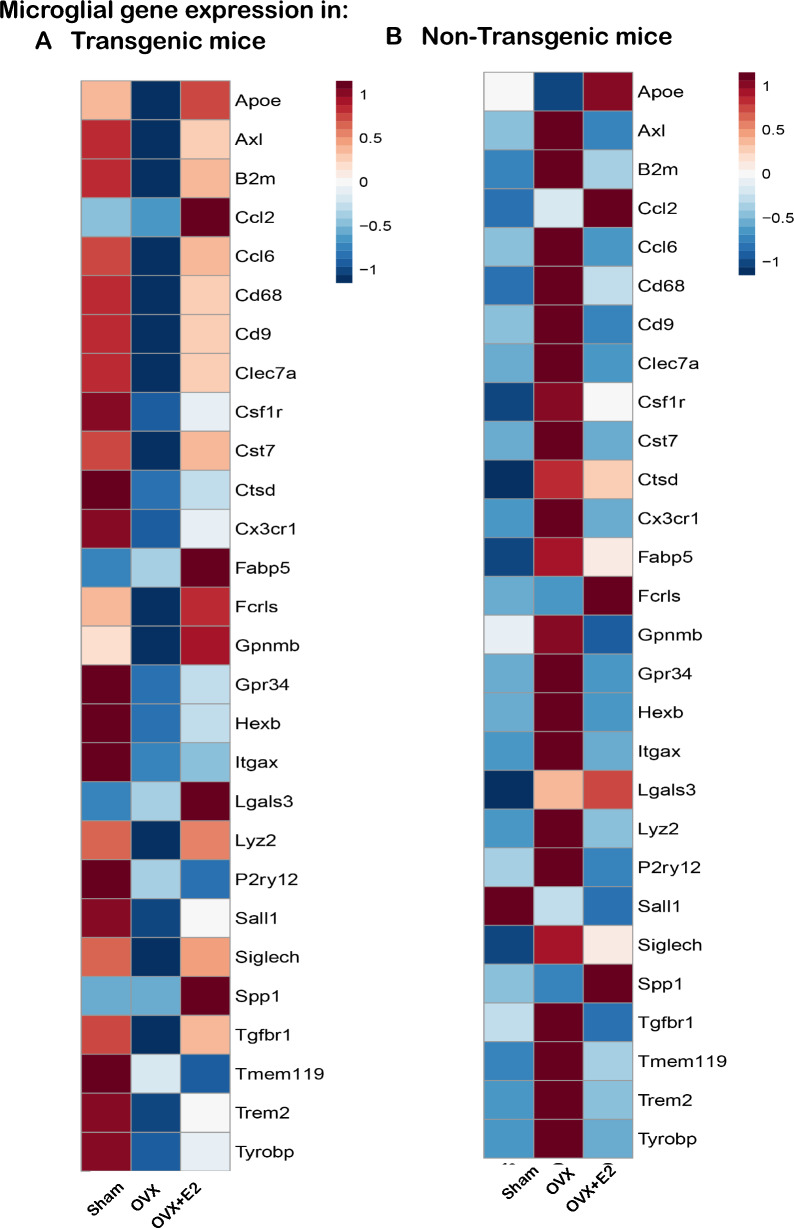
Transcriptomic analysis of microglial genes in Thy1-APPPS1 and non-transgenic mice with or without OVX and E2 supplementation. The heat map shows distinct profile variances, with significant upregulation of microglial genes in OVX non-transgenic mice, reversed by E2 supplementation (n = 6 mice/group, *p* < 0.05). An inverse trend is seen in Thy1-APPPS1 mice, except for MGnD genes P2ry12, Cx3cR1, and Hexb, which are further downregulated in the E2 supplementation cohort (*p* < 0.01). The variance emphasizes the complex interaction between microglia, estrogen levels, and amyloid-beta plaques in Alzheimer's disease.

We further examined the expression levels of a targeted panel of microglial genes through bulk RNA sequencing, as depicted in Fig. 5. The resultant heatmap analysis not only contrasted the transcriptomic profiles between transgenic APPPS1-21 mice and their non-transgenic counterparts but also revealed unexpected variations within the non-transgenic group. Notably, a marked upregulation of several microglial genes, including P2ry12, Gpr34, Tmem119, Siglech, Hexb, Csf1r, Tgfbr1, Clec7a, Lgals3, Gpnmb, Itgax, Ccl2, Fabp5, Cx3Cr1, Tyrobp, Trem2, Ctsd, B2m, Axl, Cd68, Cd9, Lyz2, and Ccl6, was observed in non-Tg mice following OVX, a trend that was distinctly reversed with E2 supplementation. These findings highlight a notable divergence in microglial-gene-specific transcriptomic profiles between non-transgenic and transgenic mice that were regulated by estradiol, as substantiated by the corresponding heatmaps (Fig. 5). The inclusion of non-transgenic mice in this study was pivotal to discern whether inherent differences in microglial composition exist between diseased and healthy states. As hypothesized, the basal microglial expression profiles were starkly different between these two cohorts.

### Differential levels of plaque-localized Clec7a + and P2y12 + microglia in OVX and estradiol-supplemented animals

**Figure 6:**
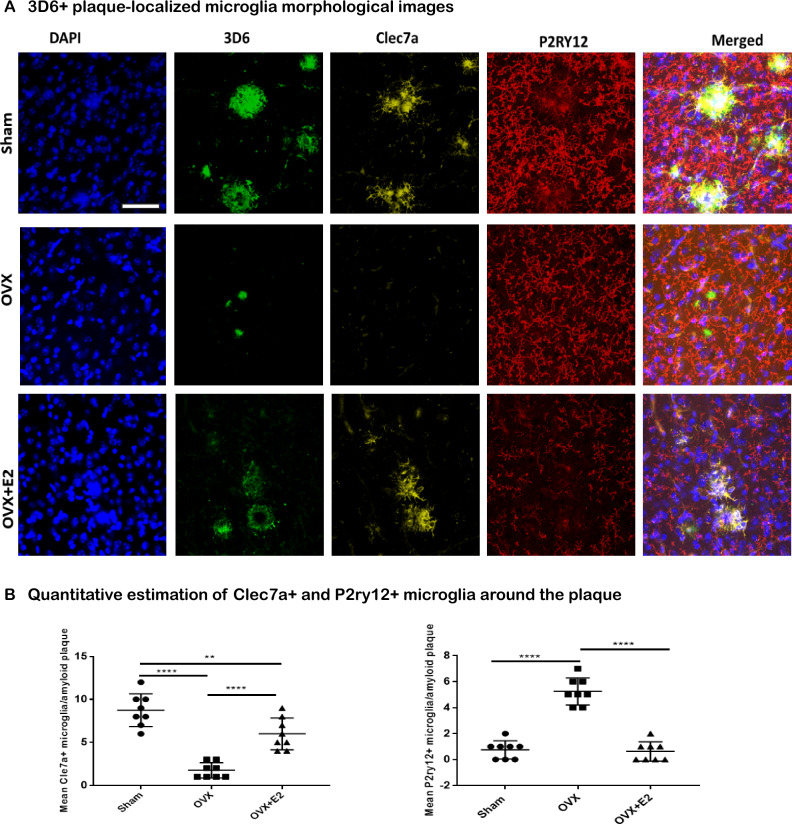
Immunohistochemical images of the effects of OVX and estradiol supplementation on plaque-localized microglial populations in APPPS1-21 mice (scale = 20 micron, n = 8 mice/group). (**A**) Immunostaining with antibodies specific for Clec7a + degenerative microglia (MGnD) and P2y12 + homeostatic microglia reveal the microglial phenotypes surrounding plaques. (**B**) Quantification shows that OVX leads to a significant decrease in Clec7a + microglia localized around plaques compared with the Sham-operated group (*p* < 0.0001, ****), while supplementation with estradiol in OVX-treated mice increases the number of Clec7a + microglia (*p* < 0.0001, ****). Conversely, OVX results in an increased number of plaque-associated P2y12 + microglia, which is significantly (*p* < 0.0001, ****) reduced with estradiol supplementation, approaching levels observed in the Sham-operated group.

To complement our transcriptomic data and clarify the spatial distribution of microglial gene expression near amyloid plaques, we performed IHC using antibodies specific for Clec7a + , that marks “MGnD”-type neurodegenerative microglia^14^, and P2y12 + , that marks M0 homeostatic microglia^14^ [Fig. 6A]. OVX resulted in a significant decrease (n = 8 mice/group, *p* < 0.001) in the number of Clec7a + microglia localized around the plaques compared with the sham-operated cohort (Fig. 6B). Conversely, mice subject to OVX and supplemented with estradiol showed a significant increase in the number of Clec7a + , plaque-associated microglia compared with the OVX cohort (n = 8 mice/group, *p* < 0.001). In contrast, OVX led to an increase in the number of P2y12 + microglia associated with plaques compared with the Sham-operated cohort. Notably, estradiol supplementation of OVX-treated mice resulted in a significant reduction in the number of plaque-associated P2y12 + microglia (n = 8 mice/group, *p* < 0.001), approaching levels similar to those observed in the Sham-operated group [Fig. 6B]. These results highlight the dynamic influence of OVX and estradiol supplementation on the microglial response in the context of plaque pathology. The modulation of microglial phenotypes by estradiol supplementation suggests a potential role for this hormone in regulating microglial function and potentially influencing neurodegenerative processes associated with plaque deposition.

## Discussion

It is now well established that the prevalence of AD in women is higher than in men^1^. Indeed, studies indicate that women who are heterozygous for APOE-ε4 are at a greater risk for developing AD than age-matched men that are heterozygous for APOE-ε4^4^. In a large meta-analysis^20^ and in a large population study of AD^21^, women who were heterozygous for the APOE-ε4 allele had AD diagnosed 5 years earlier than heterozygous men. Similarly, the odds ratio for AD in women with one copy of the APOE-ε4 allele is fourfold greater than in men^20^.

In preceding efforts, we demonstrated that postnatal perturbations of the gut microbiome with an ABX cocktail leads to a male-specific reductions in Aβ burden in both APPswe/PS1ΔE9^9,22^ and APPPS1-21 mouse models^10,19,23^ of Aβ amyloidosis. In parallel, we documented a male-specific alteration in the cortical microglial transcriptional landscape in APPPS1-21 mice^10,19,23^. With these findings, we tested the hypothesis that gut microbiome perturbations could have an influence on estrogen levels in female mice that would have an influence on Aβ burden and microglial phenotypes. We now offer several important insights that support this hypothesis. First, we document that in APPPS1-21 mice, postnatal ABX treatment leads to an elevation in circulating estradiol levels at 3 months, the time of cull. Second, OVX of young female mice at the age of 4–5 weeks [~ 2–3 weeks prior to the onset of Aβ deposition], leads to a significant reduction in Aβ burden at 3 months of age. Third, supplementation of β-estradiol in the drinking water of another cohort of mice subject to OVX restores Aβ pathology.

It is important to note that the temporal alignment of OVX with respect to the initiation of pathology is critical as this paradigm enabled us to identify early molecular and cellular events that might affect disease onset and progression. We intentionally chose neither to induce a "menopausal brain" condition by surgical removal of the ovaries after substantial pathological signs appeared nor did we instigate a chronic/protracted estradiol deprivation strategy, a method typically employed to simulate a menopausal condition in mice^24^. Additionally, we opted for a standard in-house diet instead of the phytoestrogen-free diet typically employed for chronic estrogen deprivation studies that are used to simulate a menopausal brain. Our finding that OVX performed on APPPS1-21 mice resulted in a decrease in both plaque burden and plaque size within the cortex mirrors the findings reported by Mucke and colleagues in J20-hAPP mice^25^. Moreover, when J20-hAPP mice that were subjected to OVX at 3 weeks of age were supplemented with estradiol, there was a significant increase in Aβ levels. In this latter setting, Aβ deposition nearly reverted to that observed in 2.5-month-old mice with intact ovaries^25^. In order to determine the mechanism of action[s] of estrogen on Aβ burden, we have employed transcriptomic analysis to identify the underlying biological processes that maybe affected upon ovariectomy. Our analysis disclosed that the genes most significantly affected in the cortex were predominantly associated with metabolic processes and the immune system. As microglia, the primary resident immune cells of the central nervous system, are largely implicated in amyloid clearance and are known to be metabolically driven^26^, we were motivated to investigate the status of these cells, particularly those found in the immediate vicinity of plaques. Not surprisingly, we report that the OVX-mediated reduction in amyloid burden leads to decreased levels of Clec7a + degenerative microglia and increased P2y12 + neuroprotective microglia surrounding plaques, indicating a potential neuroprotective function of lowering hormonal levels in modulating microglial responses to Aβ pathology. In contrast, estradiol supplementation reversed these effects, implying that differing levels of estradiol in the Sham, OVX, and OVX + E2 groups could selectively control microglial activation.

The finding that females subject to OVX leads to a reduction in Aβ burden was remarkable for the fact that there is very little consensus in the field on the impact of OVX in modulating Aβ burden in other mouse models of Aβ amyloidosis. For example, in the triple transgenic mouse model of AD (3xTg-AD], OVX-induced depletion of sex steroid hormones in adult female 3xTg-AD mice significantly increased Aβ accumulation and worsened memory performance; treatment of ovariectomized 3xTg-AD mice with estrogen, but not progesterone, prevented these effects^27^. On the other hand, Heikkenen and colleagues^28^ reported that in transgenic mice that coexpress FAD-linked APPswe and PS1-A246E, OVX or estrogen supplementation of OVX or sham-operated mice had no effect on hippocampal amyloid accumulation. Similarly, Green et al. (2005) reported that in female PDAPP mice that express the FAD-linked APP V717F mutation driven by the PDGF promoter, neither OVX nor OVX with estrogen replacement altered Aβ accumulation in the hippocampus or neocortex. However, Zheng et al.^24^ reported that OVX of Tg2576 mice that express APPswe driven by the mouse PrP promoter, resulted in a significant increase in the level of soluble Aβ40 compared with sham-operated littermates and a trend towards increases in Aβ42 peptides, but these increases in Aβ peptides were not associated with an increase in Aβ deposition. In contrast, Levin-Allerhand and colleagues^29^ reported that OVX in AβPPSWE transgenic mice did not alter Aβ levels, but that the levels of Aβ were decreased by 17β-estradiol. Mechanistically, it was suggested that estradiol increased the levels of sAβPPα, and hence, the ratio of Aβ/sAβPPα, a measure of amyloidogenic processing, was reduced in all estrogen-containing groups. It is important to note that all of the previous studies did not typically account for variations in the estrus cycle, which now casts a shadow of doubt over the results. On the other hand, in a recent investigation where the estrus cycle was considered, a transient spike in estradiol lead to an increase in Aβ1-42 levels, while OVX was shown to decrease the levels of this peptide in J20-hAPP mice^25^. Complementing these findings, research has also shown that fluctuating estrogen levels during pregnancy are linked to an increased risk of AD. This risk appears to be further amplified in cases of multiple pregnancies (multiparous)^31^. In summary, a consensus view on the impact of OVX on plaque pathology or associated cellular/biochemical outcomes in transgenic mouse models has not emerged. These variations across studies may be due to differences in the specific animal models used, the age of the animals, the exact timing of hormone and Aβ measurements within the proestrus stage, and other methodological differences.

Given the well-documented risk factors, empirical studies spanning two decades had posited estrogen therapy as a mitigative strategy against cognitive decline, specifically targeting a proposed 'critical window' at approximately 55 years of age in females^30^. This long-standing assumption has been decisively challenged by a recent Danish case–control study (Pourhadi et al., 2023) conducted between 2000 to 2018 that leveraged a comprehensive dataset that included 5589 dementia cases and 55,890 controls. The data conclusively show that estrogen therapy is associated with a heightened risk for all forms of dementia and Alzheimer's-type dementia. Notably, this increased risk is independent of the duration of therapy or the age at which it is initiated. These compelling results mandate an immediate and comprehensive reevaluation of the previously assumed benefits of estrogen therapy in women. In this regard, the estrus cycle—a reproductive cycle in female mammals typified by hormonal oscillations—has been largely disregarded in the domain of AD research^31^. Historically, AD studies have either chiefly concentrated on male subjects or insufficiently acknowledged the impact of the estrus cycle in female subjects^32^. This oversight may have significant implications for our understanding of the disease and the development of effective treatments. Emerging evidence suggests that the estrus cycle indeed influences AD pathology and cognitive function in animal models^25^. Studies using transgenic mouse models of AD have shown that hormonal fluctuations during the estrus cycle can impact Aβ accumulation, tau pathology, neuroinflammation, and synaptic function^25,33,34^. For example, higher levels of estradiol during specific phases of the estrus cycle have been associated with increased Aβ burden in J20-hAPP mice^25^. These findings suggest that the hormonal changes associated with the estrus cycle may have a critical window of opportunity for influencing AD pathogenesis. Neglecting the estrus cycle may lead to inconsistent or misleading results, as hormonal variations could mask or confound the effects of experimental interventions. Therefore, while there is some evidence suggesting that the proestrus phase may be linked with elevated levels of Aβ^25^, we maintained consistency in our study by sacrificing all animals during/at the proestrus stage.

Finally, a critical factor to consider is that the microbiome composition of different animal facilities can vary significantly^35,36^, and these differences can have implications for research outcomes, including studies on OVX in AD mice models. In our study, we observed that administering exogenous estradiol to the OVX group did not mirror the microbiome composition of the Sham group. This outcome stemmed from our approach of modulating the microbiome's growth and composition by providing exogenous estradiol in the drinking water for about five weeks. This method impeded the natural growth and recolonization of microbes, leading to a microbiome composition distinct from the natural one in the Sham group at the time of cull. These findings underscore the critical influence of hormonal intervention on microbiome dynamics and highlight the intricate complexities inherent in such experimental settings, emphasizing the nuanced interplay between hormonal treatments and microbial ecosystems in scientific research. Additionally, the microbiome, consisting of the community of microorganisms residing in and on the body, has been recognized as a critical factor influencing various aspects of health and disease, including brain function and neurodegenerative disorders, including AD^37^. Animal facilities maintain specific environmental conditions, including housing, diet, and microbial exposure, which can shape the microbiome of the animals housed within them^38,39^. Therefore, when studying the effects of OVX in AD mice models, variations in microbiome composition across different animal facilities may influence the outcomes of these studies. Moreover, it is essential to replicate findings across different animal facilities to validate the robustness and generalizability of the observed effects of OVX in AD mice models. Collaborative efforts and multi-center studies that involve multiple animal facilities can provide a more comprehensive understanding of the role of estrogen in AD and the impact of the microbiome.

Our study offers valuable insights into the interplay between estrogen, gut microbiome perturbations, metabolic alterations and Aβ burden in female mouse models of Alzheimer's disease. However, there are several limitations of our studies that need to be addressed in future research. First, our study largely relies on a specific mouse model, raising questions about the generalizability of the findings to other models or to humans. The variability in the outcomes of OVX on Aβ burden across different animal models, as noted above, underscores this concern. Second, while our study intentionally avoided inducing a "menopausal brain" condition in mice, this choice limits our understanding of how menopause may impact AD. Third, the effects of ovariectomy were primarily examined in the context of estradiol, without considering the potential roles of other sex hormones like progesterone. Lastly, our study was conducted in a single animal facility, and the role of different microbiome compositions across various facilities on the outcomes has not been addressed. Future studies should aim to address these limitations, perhaps by incorporating a broader range of animal models, including menopausal conditions, examining the roles of other sex hormones, and collaborating across multiple animal facilities to verify the robustness and generalizability of the findings.

In summary, our research delineates the multifaceted interplay among estrogenic modulation, gut microbiome dysregulation, metabolic shifts, and Aβ accumulation within the context of female murine models of Aβ amyloidosis. These findings provide valuable insights into underlying mechanisms and offer new avenues for the design of preventive and therapeutic interventions that target hormonal and microbial factors involved in the modulation of Aβ amyloidosis and neuroinflammation.

## Materials and methods

### Animal handling and housing

APPPS1-21 mice, acquired from M. Jucker at the University of Tubingen, Germany, were raised on a C57BL6Cj background. The mice were kept in sterile micro-isolator cages and provided with standard chow ad libitum. All procedures involving animal experimentation were conducted following approved Animal Care and Use Protocols by the Institutional Animal Care and Use Committee at the University of Chicago. We hereby confirm that this study has been reported in accordance with the ARRIVE (Animal Research: Reporting of In Vivo Experiments) guidelines, as outlined at https://arriveguidelines.org.

### Antibiotics treatment

Pups given the ABX were administered orally (200 μl ABX via animal feeding syringes; item number 7901; Cadence) with a predefined ABX mixture (Stefka et al., 2014; 4 mg/ml kanamycin, Sigma-Aldrich K4000-5g; 0.35 mg/ml gentamicin, Sigma-Aldrich G1914-250mg; 8500 U/ml colistin, Sigma-Aldrich C4461-1g; 2.15 mg/ml metronidazole, Sigma-Aldrich M1547-25g; 0.45 mg/ml vancomycin, Sigma-Aldrich V2002-1g) in sterilized water from day P14 to P21. Throughout the 7-day postnatal ABX administration, mice were moved to a clean, germ-free cage after each dosage to prevent bacterial exposure from cage droppings. Adult mice from the same cage as the ABX-treated pups were put down post-weaning and were excluded from further experiments or breeding. The ABX water was refreshed every 5–6 days.

### Necropsy and tissue harvesting

The procedure for harvesting various organs from mice at the time of sacrifice was conducted according to the approved and published Animal Care and Use Protocols of the University of Chicago. On the day of euthanasia, mice were given an intraperitoneal injection containing a mixture of ketamine and xylazine. After confirming deep anesthesia, blood samples were drawn through cardiac puncture using a 25-gauge needle, then stored in EDTA-coated collection tubes (BD Vacutainer; 365974) and placed on ice. Immediately following blood collection, the descending aorta was clamped, and the mice were perfused with physiological cold saline (pH 7.4) for 3 min. The brains were then removed from the skulls and divided into two hemispheres: one was postfixed with 4% paraformaldehyde for histological examination, and the other was immediately frozen for transcriptome analysis. The cecum was collected and weighed to assess the efficacy of the antibiotic treatments. Upon completion of these procedures, plasma was separated by centrifuging at 2000 rpm for 10 min at 4 °C using a Beckman Coulter centrifuge and stored at − 80 °C for future use.

### GC–MS Analysis of Estrogen Metabolites in Mouse Serum

For mass spectrometry analysis, 500 µL of serum was dried down under nitrogen to completion. To improve the volatility of estradiol and estrone, the metabolites were derivatized prior to analyses. To the sample vial, 250 µL of N,O-Bis(trimethylsilyl)trifluoroacetamide (BSTFA) and 250 µL of pyridine were added and incubated in a thermomixer at 70 °C for 30 min at 1400 rpm. Samples were then dried down under nitrogen to completion and reconstituted with 1 mL of hexane. Following derivatization, 1 µL of each sample was injected into a gas chromatography-mass spectrometer (GC–MS, Agilent 7890B GC/ Agilent 5977B MSD). Metabolites were separated (HP-5MSUI column, 30 m × 0.25 mm, 0.25 µm) and detected using GC–MS with electron impact (EI) ionization with helium as the carrier gas. The oven temperature was held at 200 °C for 1 min, increased 10 °C/min from 200 to 250 °C, then increased 5 °C/min from 250 to 270 °C, and finally increased at 10 °C/min from 270 to 300 °C. Data analysis was performed using MassHunter Quantitative Analysis software (version B.10, Agilent Technologies) and confirmed by comparison to authentic standards and a similarity search of the NIST Tandem Mass Spectral Library Version 2.3. The retention time and fragmentation pattern of derivatized authentic standards were used to verify endogenous serum compounds.

### Experimental models

#### Ovariectomy

Ovariectomy was performed at 5 weeks of age and sham surgery was performed on littermate animals. The animals were scarificed at 3 months of age, brains were removed for analysis and uterine weights were assessed.

#### Estradiol supplementation study

For estrogen supplementation, female mice were ovariectomized at 5 weeks of age. Starting at 8 weeks of age, estradiol was administered in the drinking water, following the protocol established by Gordon et al. (1986)^40^. Estradiol (Sigma, St. Louis, MO, USA) was first dissolved in ethanol to create a stock concentration of 5 mg/ml. This stock solution was then aliquoted into fresh drinking water to achieve a working concentration of 5 µg/ml. To maintain this concentration consistently throughout the experimental period, careful measures were taken, including accurate measurement of the solution, regular monitoring of drinking water levels, and timely replacement of the solution as needed.

#### ELISA of hormones

Serum levels of E_2_ and P_4_ were measured at the proestrus stage. Blood was collected under anesthesia at 0800 h of the corresponding day by cardiac puncture, and serum was procured after centrifugation at 3000 g for 5 min and stored at – 80 °C until assayed. E_2_ levels were assayed using a commercial ELISA kit from ALPCO (11-ESTHU-E01; Alpco, Salem, NH), while P_4_ levels were measured with an automated chemiluminescence assay system (Immulite 2000; Diagnostics Products Corp., Los Angeles, CA, USA). The minimal detection limits for these assays were 0.08 ng/ml for P4 and 5 pg/ml for E2, with both intra- and inter-assay coefficient of variations being 10% for each assay.

#### Cerebral cortex RNA extraction, library preparation and sequencing

Total RNA was extracted from the dorsal cerebral cortex using Trizol reagent, as per the protocol by Dodiya et al.^10^, and cleaned with the RNAeasy Micro kit (Qiagen). Quality assessment was done with an Agilent Bioanalyzer. RNA-seq library preparations and sequencing were performed using an Illumina HiSeq4000 at The University of Chicago Genomics Core Facility. The data were collected in FASTQ format for bioinformatic analysis.

#### RNA-seq bioinformatics analysis

The quality of DNA reads, provided in FASTQ format, was assessed using FastQC. Adapters were trimmed, and reads that were either of poor quality or aligned to rRNA sequences were filtered using Trim Galore (http://www.bioinformatics.babraham.ac.uk/projects/trim_galore/). The cleaned reads were then aligned to the mouse genome (mm10) using STAR. Read counts for each gene were computed using HTSeq-Counts, in conjunction with a gene annotation file for mm10 obtained from Ensembl (http://useast.ensembl.org/index.html). A comprehensive quality control report was assembled using MultiQC. Differential expression was analyzed using DESeq2, with a cut-off for identifying significant differentially expressed genes (DEGs) set at an FDR-adjusted *P* value of < 0.05. Gene Ontology (GO) analysis and identification of DEGs within specific pathways were conducted using Metascape, and the findings were validated using the DAVID and Gorilla online services. The data discussed in this publication have been deposited in National Center for Biotechnology Information’s Gene Expression Omnibus and are accessible through GEO Series accession no. GSE245831 (https://www.ncbi.nlm.nih.gov/geo/ query/acc.cgi?acc = GSE245831).

#### Fecal DNA extraction

Bacterial DNA was extracted from cecal and fecal contents following the method previously described and in accordance with standardized Earth Microbiome Project protocols (www.earthmicrobiome.org/emp-standard-protocols/). In brief, tissues were dissolved in an extraction buffer (50 mg tissue/ml buffer, 50 mM Tris (pH 7.4), 100 mM EDTA (pH 8.0), 400 mM NaCl, 0.5% w/v SDS) containing 0.4 mg/ml of proteinase K. Upon the addition of glass beads with a diameter of 0.1 mm (500 µl/ml buffer, BioSpec Products), microbial cells were lysed using a Mini-Beadbeater-8K Cell Disrupter (BioSpec Products), followed by overnight incubation in a water bath at 55 °C. Total DNA was subsequently extracted using a Phenol:Chloroform:IAA mixture (25:24:1 v/v, pH 8.0, Ambion), following the manufacturer's guidelines. The DNA yield was quantified, and its quality was determined using a combination of Nanodrop Lite (Thermo Fisher) and Qubit® fluorometer (Invitrogen) assessments.

#### 16S rRNA gene Illumina® MiSeq sequencing

The 16S rRNA gene copy number was quantified from DNA isolated from fecal and cecal contents using quantitative PCR (QPCR). A concentration of 2–5 ng/µl DNA was combined with iQ-SYBR Green PCR Supermix (Bio-Rad), 518F (5′-TCC-TAC-GGG-AGG-CAG-CAG-T-3′) and 338R (5′-GGA-CTA-CCA-GGG-TAT-CTA-ATC-CTG-TT-3′) primers (2.5 μM), and Q-PCR was carried out on a LightCycler® 480 system (Roche). The reaction conditions were as follows: an initial denaturation at 95 °C for 5 min, followed by 35 cycles of 95 °C for 10 s, 64 °C for 45 s, and 72 °C for 45 s, and a final extension at 40 °C for 30 s. The 16S rRNA gene copy number was determined by referencing Cp values to a standard curve created from the pCR4-TOPO plasmid containing the 16S rRNA gene amplicon. The copy number was then expressed relative to the specific DNA concentration added per reaction, as determined by a prior assessment using a Qubit® fluorometer (Invitrogen). All reactions were conducted in triplicate and included appropriate negative controls. The datasets generated and/or analyzed during the current study are available in the National Center for Biotechnology Information (NCBI) Sequence Read Archive (SRA) under accession number PRJNA1030000 (https://www.ncbi.nlm.nih.gov/bioproject/PRJNA1030000).

#### Fecal microbiota analysis

We utilized the Quantitative Insights Into Microbial Ecology (QIIME) pipeline and vsearch 8.1 to conduct standard demultiplexing and quality filtering. Unique ASVs were identified utilizing the Deblur method, and taxonomy was assigned through the Greengenes Database (May 2013 release; http://greengenes.lbl.gov). Statistical analyses were carried out in R, relying primarily on the 'vegan' and ‘phyloseq’ libraries.

#### Western blot analysis

Brain lysates were prepared from Thy1-APP/PS1 hemibrains using a lysis buffer composed of 50 mM Tris (pH 7.4), 150 mM NaCl, 5 mM EDTA, 0.5% NP-40, 0.5% sodium deoxycholate, 1 × protease inhibitor cocktail, 1 mM PMSF, and 1 × phosphatase inhibitor cocktail. Post-homogenization and sonication on ice, samples were centrifuged at 10,000 g for 20 min at 4 °C. The supernatant was then collected as the total protein lysate. Protein concentrations were quantified using BCA analysis. For gel electrophoresis, samples were denatured at 95 °C in reducing buffer prior to running on 12% glycine SDS-PAGE or 10–16.5% tricine SDS-PAGE gels, as described in Minter et al., 2016. This was followed by a wet transfer to a 0.2 μm nitrocellulose membrane. The membranes were blocked in 5% non-fat milk in PBS-T for 1 h at room temperature. For antibody detection, Human FL-APP was identified using a 6E10 antibody (Biolegend) at a 1:2000 dilution. β-Actin was employed as a loading control, detected with an Actin primary antibody at a 1:15,000 dilution. All blots used an anti-mouse secondary antibody at a 1:5000 dilution. After washing, the membranes were incubated with HRP-conjugated secondary antibodies for 1 h. Signal detection was carried out using Western Lightning® Plus ECL and X-ray film. Densitometry analysis was performed using ImageJ.

#### MSD analysis

To measure both soluble and insoluble Aβ levels, we used the ventral halves of frozen brains stored at – 80 °C. These halves were homogenized in a TBS solution with protease inhibitors and EDTA. After ultracentrifugation, the supernatant was collected for soluble Aβ analysis. The remaining pellet was then processed in 70% FA and ultracentrifuged again to collect the supernatant for insoluble Aβ levels. These samples were sent to Harvard University for MesoScale Aβ analysis, performed according to protocols by Minter et al. (2016) using the Quickplex SQ 120 system.

#### Immunocytochemistry

To assess Aβ amyloidosis, immunofluorescence staining was performed following a previously published protocol. Specifically, a complete series of 40 μm-thick brain sections were stained for Aβ (3D6, 1:10,000)^9^, while a half series was used for microglia staining with primary antibodies P2RY12 (Invitrogen, 1:1000) and Clec7a (Invitrogen, 1:250). The procedure began with washing free-floating, level-matched 40-μm sections in dilution media, with a duration of 60 min (10 min per wash). Sections were then immersed in serum blocking solution for an hour at ambient temperature, succeeded by overnight incubation with primary antibodies at 4 °C. The subsequent day, the sections underwent a 60 min wash in dilution media (10 min/wash), followed by a one-hour incubation with secondary antibodies at room temperature. Donkey anti-mouse 488 (Invitrogen; 1:500), donkey anti-rabbit 647 (Invitrogen; 1:500) and donkey anti-rabbit 647 (Invitrogen; 1:500) were employed as secondary antibodies for Aβ and microglia staining, correspondingly. Afterward, sections were washed, mounted on glass slides, and coverslipped using Fluoromount aqueous mounting medium (Sigma-Aldrich; F4680). The images of 3D6 + Aβ plaques were obtained with a 3D Histech Pannoramic MIDI whole-slide scanner and a Zeiss AxioCam MRM CCD camera, handled by personnel from The University of Chicago Integrated Light Microscopy Facility. Meanwhile, microglia images were taken utilizing a Leica SP5 3D STED laser-scanning confocal microscope at 40 × 1.5 magnification.

#### Abeta burden analysis

Evaluation of Aβ burden and amyloid plaque size was conducted in accordance with a previously defined protocol^9,10,19,22^. Briefly, six equidistant sections at 480 μm intervals, ranging from beyond the olfactory bulb to the mid-hippocampal level, were employed for the analysis of 3D6 + amyloid plaques. The Fiji ImageJ software (NIH; ImageJ 1.51n) was used by two independent and blinded observers to determine the average plaque size and Aβ fraction area, also known as the amyloid burden. Individual images were processed using the 3D Histech Pannoramic viewer software (3DHistech Kft), and subsequently normalized. Automated thresholding, based on histogram entropy, was applied to identify the amyloid plaques. After converting the images into an 8-bit format, a specific threshold number was applied, and this was followed by the use of the “fill holes” and “watershed” algorithms for binary conversion. Finally, the plaque number, plaque size area, and total area encompassed by plaques (i.e., Aβ burden) were computed using the “analyze particles” function. The Aβ burden, represented as the area fraction, was calculated by dividing the total area occupied by plaques by the total area of the cerebral cortex, which was the region of interest. Graphical representations were created utilizing the data pertaining to both amyloid burden and plaque size.

#### Statistical analysis

Statistical analyses were carried out using the GraphPad Prism software, version 7.0e. For comparing the α- and β-diversity indices in the 16S rRNA microbiota analysis across multiple groups, the Wilcoxin analysis was utilized. This allowed for the careful comparison of distributions and medians among different groups, and corrections were made for multiple comparisons to ensure the integrity of the results. For other studies, such as short-term ABX studies and comparisons between Sham, OVX and OVX + E2 studies, one-way ANOVA (Analysis of Variance) was employed, unless otherwise specified in the methodology. The one-way ANOVA enabled the examination of differences among means for several groups, and it was followed by post hoc comparisons to identify which specific groups were different from each other. In all the analyses, a statistical P value below 0.05 was considered indicative of significant differences between the groups under comparison. Further details and specifics regarding the statistical methods and findings, including any additional tests or adjustments that were made, can be found in the figure legends accompanying the results.

### Supplementary Information

Supplementary Legends.

Supplementary Figure 2.

Supplementary Figures.

## Data Availability

The datasets generated and/or analyzed during the 16S rRNA gene Illumina® MiSeq sequencing are available in the National Center for Biotechnology Information (NCBI) Sequence Read Archive (SRA) under accession number PRJNA1030000 (https://www.ncbi.nlm.nih.gov/bioproject/PRJNA1030000). The data discussed in the RNA sequencing analysis have been deposited in National Center for Biotechnology Information’s Gene Expression Omnibus and are accessible through GEO Series accession no. GSE245831 (https://www.ncbi.nlm.nih.gov/geo/ query/acc.cgi?acc = GSE245831).
