# Supplementary material for: Early modulation of the gut microbiome by female sex hormones alters amyloid pathology and microglial function
Source: Sci Rep. 2024 Jan 21;14:1827. doi: 10.1038/s41598-024-52246-6 (PMC10800351; doi:10.1038/s41598-024-52246-6)
Supplement: Supplementary file 1 — Supplementary Legends. [file 41598_2024_52246_MOESM1_ESM.docx]

**Figure 2S:** (A) Validation of Successful OVX in APPPS1-21 AD Female Mice: (a) Body weight significantly increases with OVX and is reversed with estradiol supplementation (p < 0.05*, n=12 mice/group). (b) Uterine weight decreases in OVX mice and increases with estradiol supplementation (n=7-8 mice/group, p < 0.05*, p < 0.01**). (c) Circulating estradiol and progesterone levels drop in OVX mice and are reversed with estradiol supplementation (n=4-5 mice/group, p < 0.05*). These results validate the successful execution of ovariectomy in mice, reflecting its impact on key physiological parameters. Data are represented as mean ± SEM; *, p < 0.05; **, p < 0.01. (B) Analysis of Human APP751 Expression in OVX and Estradiol-Supplemented Female APPPS1-21 AD Mice (n=3/group) Using Western Blot: Cortical extracts were analyzed using Western blot with an APP-specific C-terminal antibody. The bar graph represents the quantification of the blot using ImageJ. No significant impact of OVX or estradiol on APP751 levels in the cortex was observed. Data are represented as mean ± SEM.
