# Supplementary figures and images for: Early modulation of the gut microbiome by female sex hormones alters amyloid pathology and microglial function
Source: Sci Rep. 2024 Jan 21;14:1827. doi: 10.1038/s41598-024-52246-6 (PMC10800351; doi:10.1038/s41598-024-52246-6)

A Verification of ovariectomy surgery

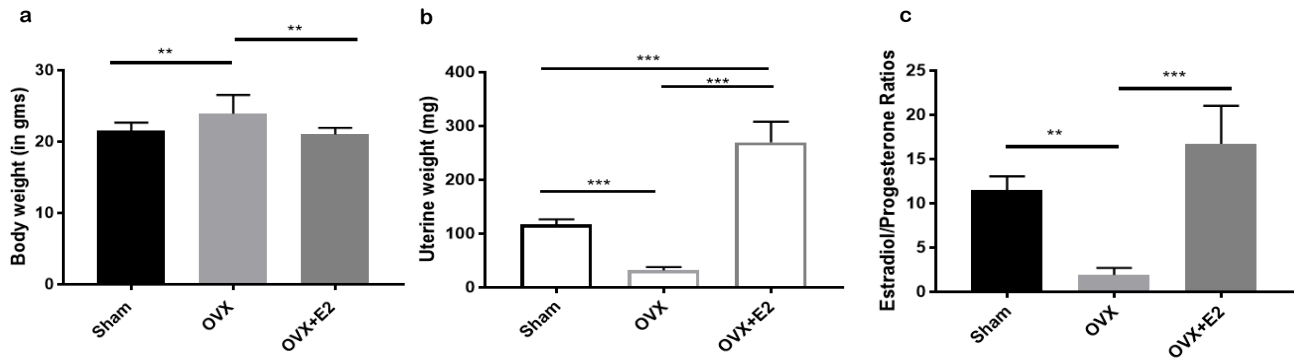

B Western blot to confirm APP full length

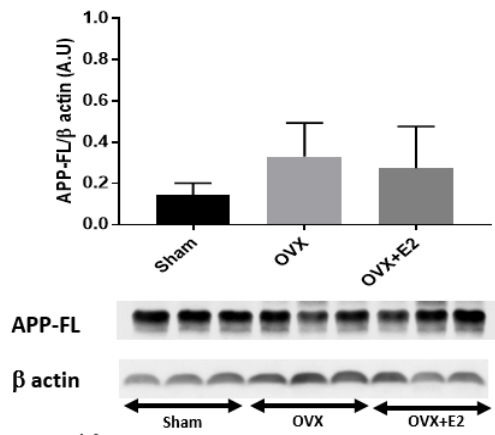

Supplement: Supplementary file 2 — Supplementary Figure 2. [file 41598_2024_52246_MOESM2_ESM.pdf]
