# Supplementary material for: Early modulation of the gut microbiome by female sex hormones alters amyloid pathology and microglial function
Source: Sci Rep. 2024 Jan 21;14:1827. doi: 10.1038/s41598-024-52246-6 (PMC10800351; doi:10.1038/s41598-024-52246-6)

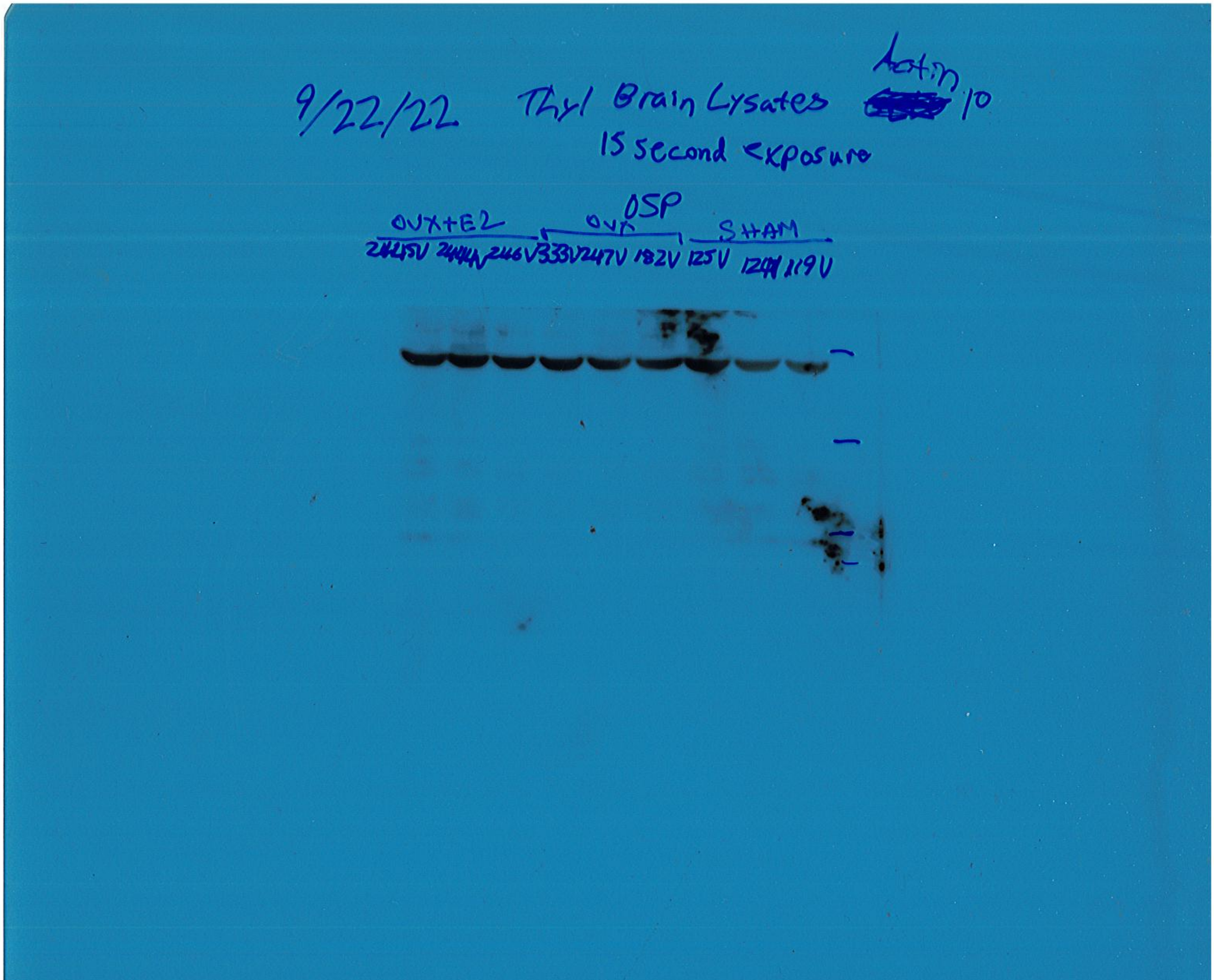
9/22/22
Thy1 Brain Lysates [illegible] Actin 10
15 second exposure
OSP
OVX+E2
OVX
SHAM
2445V 2444V 246V 333V 247V 182V 125V 124V 119V
STRATAGENE

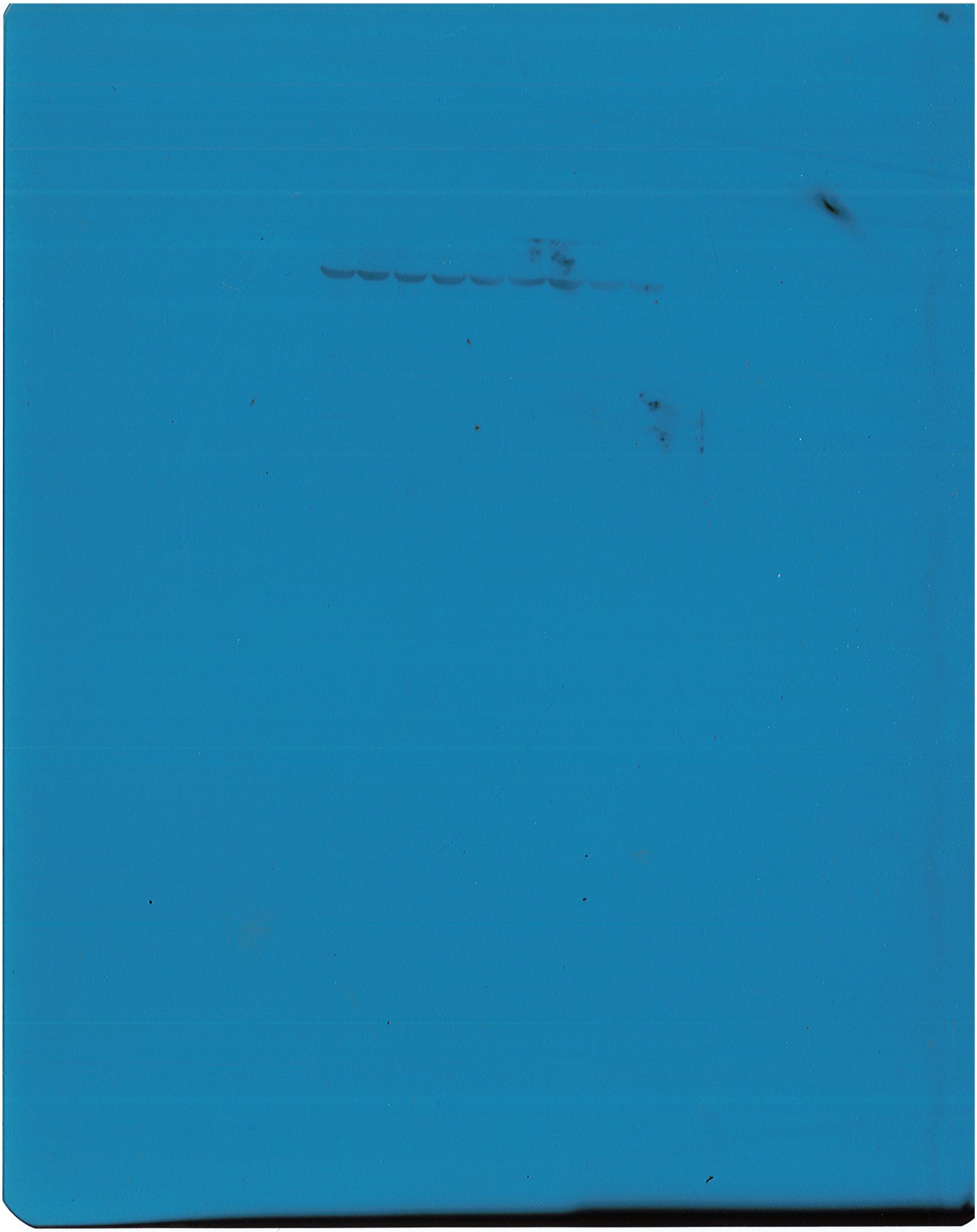

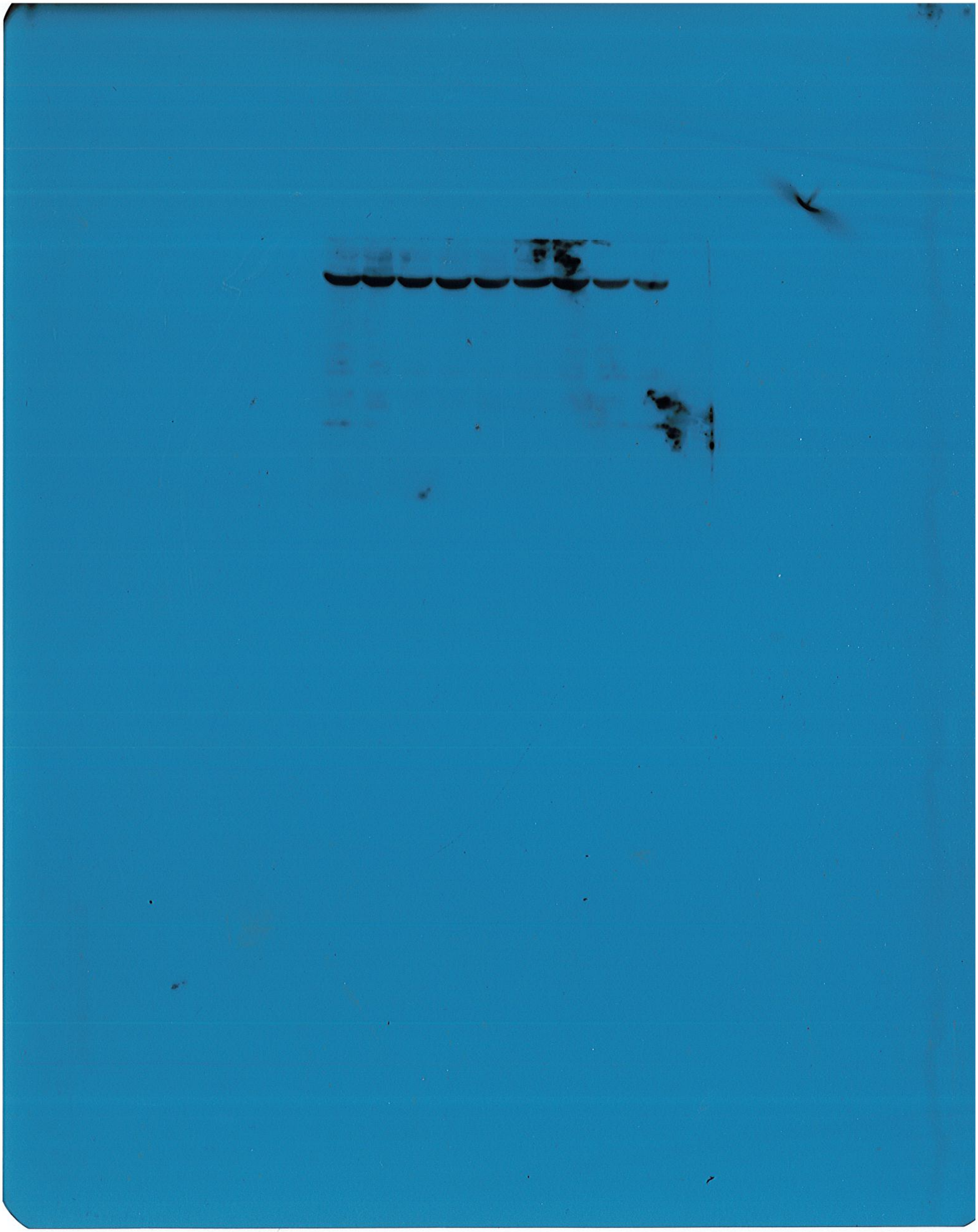

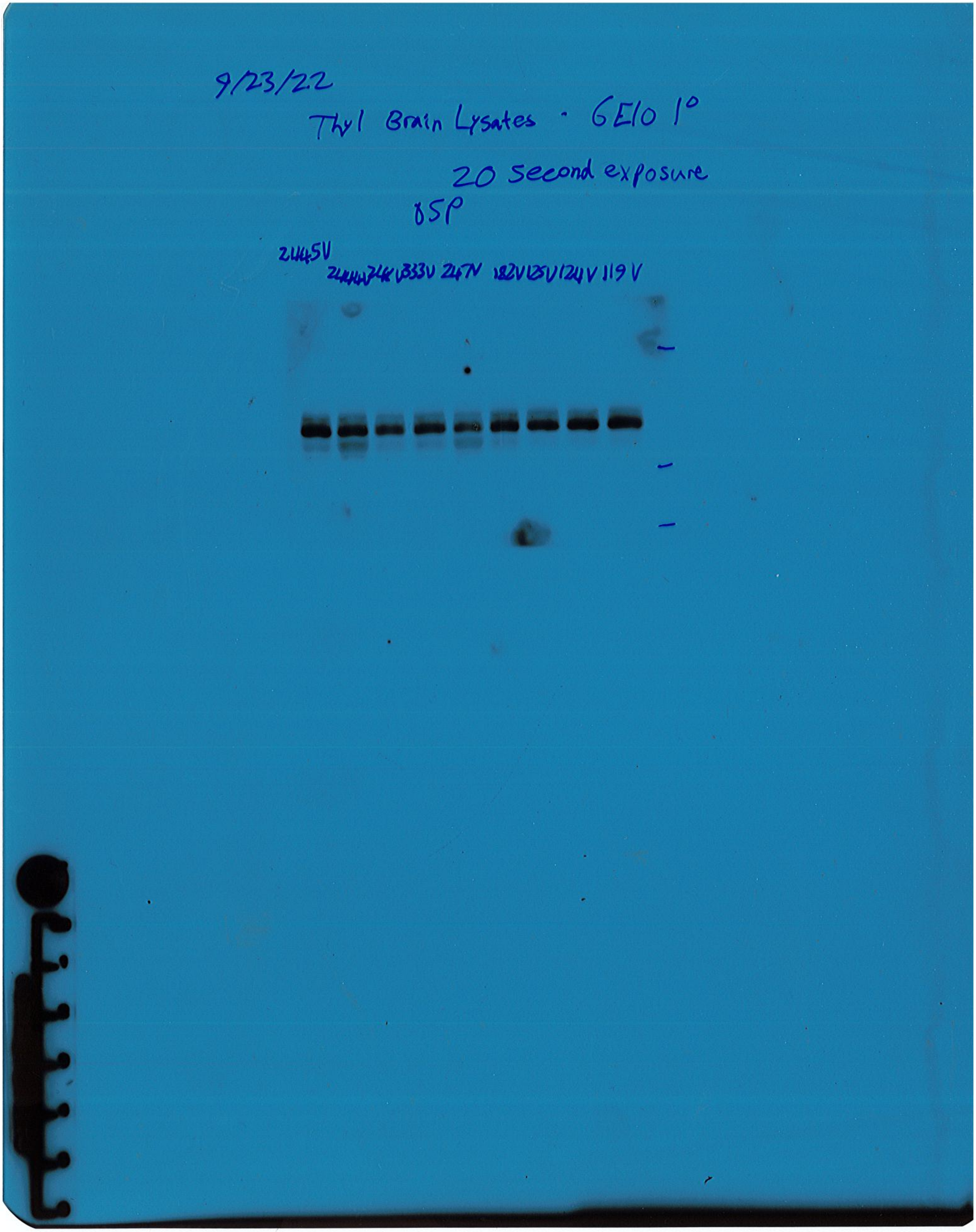
9/23/22
Thy1 Brain Lysates · 6E10 1°
20 second exposure
85P
2445V
[illegible] 333V [illegible] 182V 125V 124V 119V

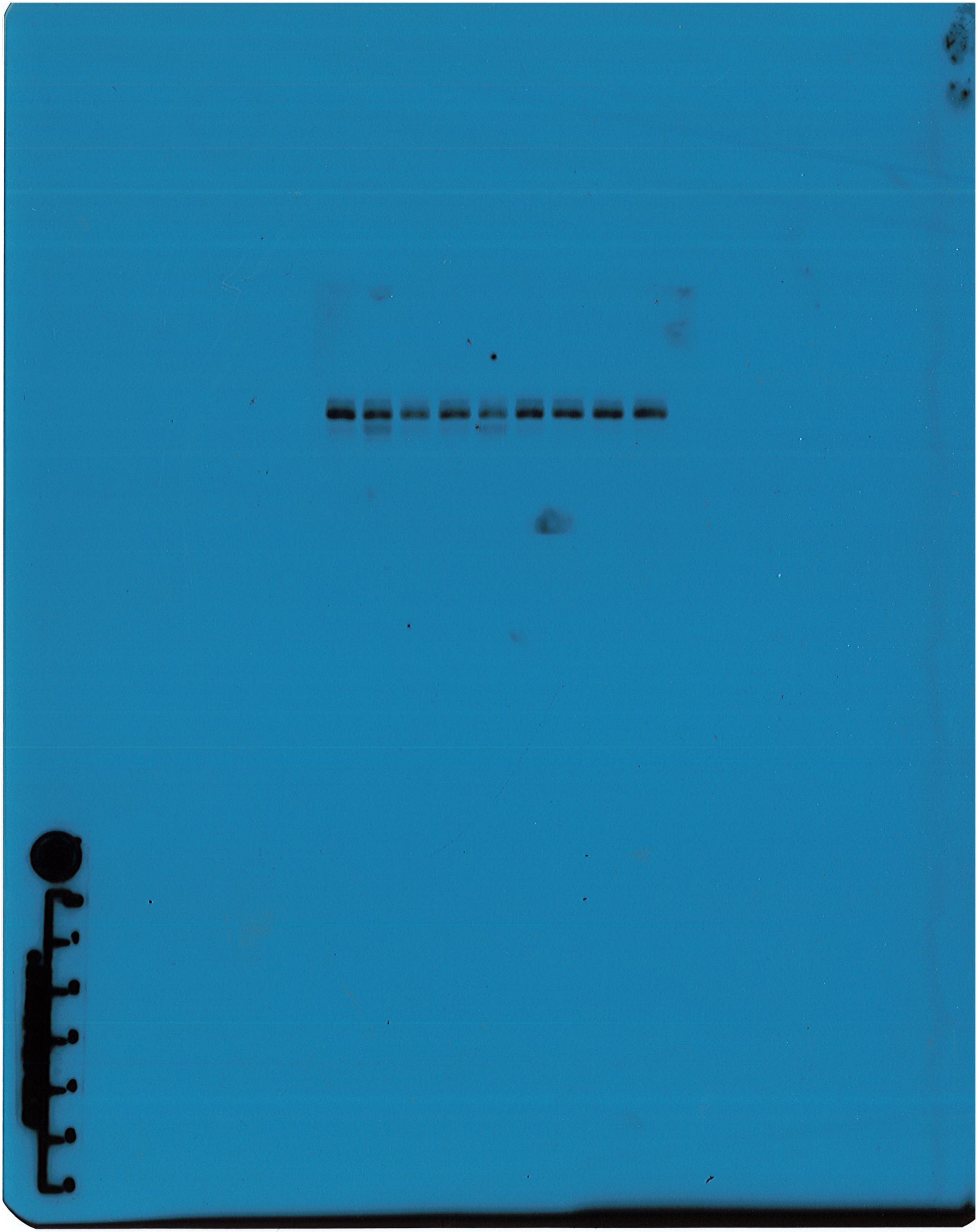

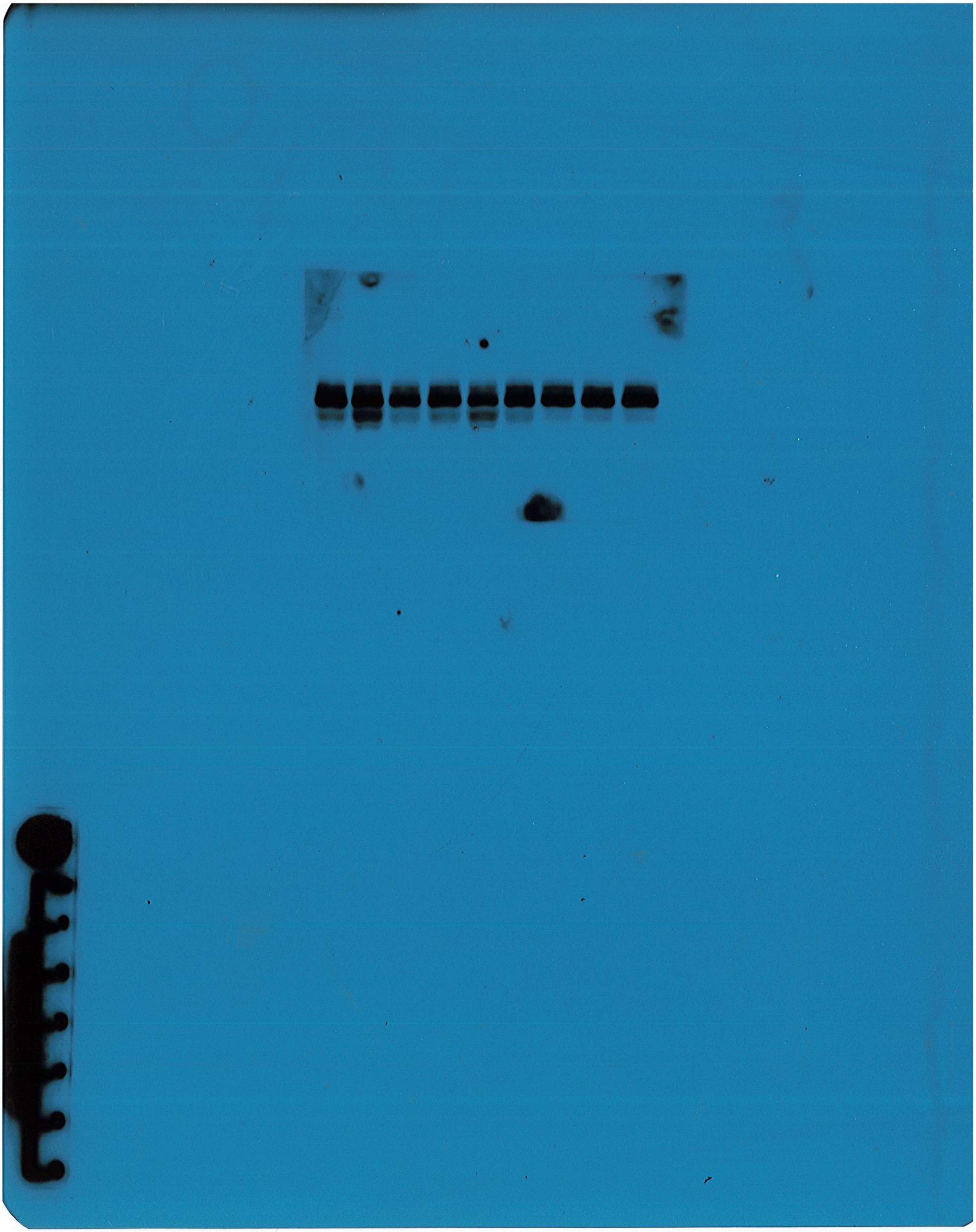

Supplement: Supplementary file 3 — Supplementary Figures. [file 41598_2024_52246_MOESM3_ESM.pdf]
